# Dr. Yang Zhong: An explorer on the road forever

**DOI:** 10.1007/s13238-017-0496-1

**Published:** 2017-12-18

**Authors:** Fan Chen, Bao-Rong Lu, James C. Crabbe, Jia-yuan Zhao, Bo-jian Zhong, Yu-peng Geng, Yu-fang Zheng, Hong-yan Wang

**Affiliations:** 1 Institute of Genetics and Developmental Biology, Chinese Academy of Sciences, Beijing, 100101 China; 2 Ministry of Education, Key Laboratory for Biodiversity Science and Ecological Engineering, Department of Ecology and Evolutionary Biology, Fudan University, Shanghai, 200438 China; 3 Wolfson College, University of Oxford, Linton Road, Oxford, OX2 6UD UK; 4 Department of Life Sciences, Institute of Biomedical and Environmental Science & Technology, University of Bedfordshire, Park Square, Luton, LU1 3JU UK; 5 College of Life Sciences, Nanjing Normal University, Nanjing, 210046 China; 6 School of Ecology and Environmental Science, Institute of Ecology and Geobotany, Yunnan University, Kunming, 650091 China; 7 Obstetrics & Gynecology Hospital, Institute of Reproduction & Development, Fudan University, Shanghai, 200090 China

**Figure 1:**
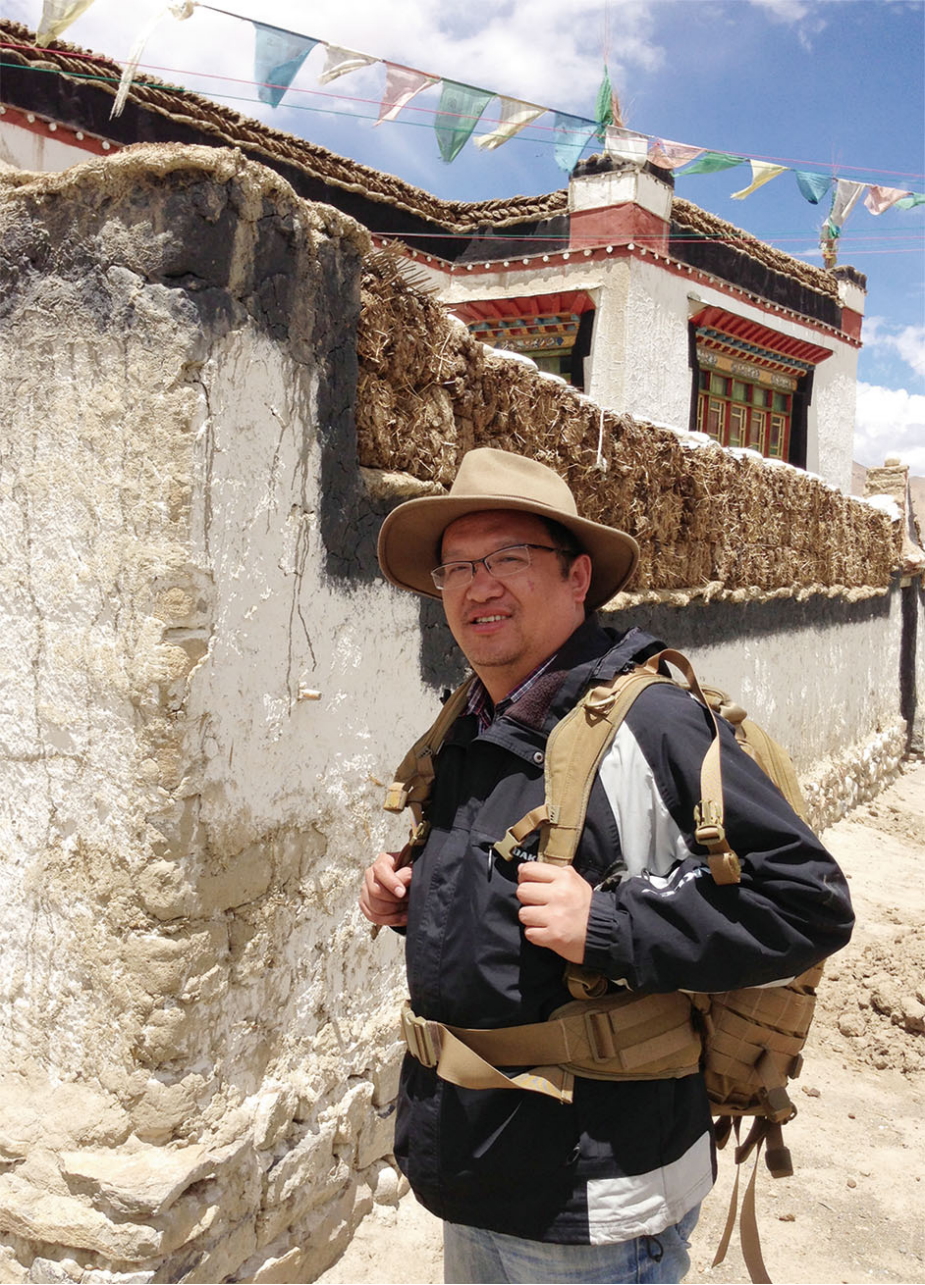
**Dr. Yang Zhong: An explorer on the road forever**

On the morning of September 25th 2017, grievous news spread from the remote Ordos region of Inner Mongolia to Fudan University campus in Shanghai. Professor Yang Zhong (Fig. 1), a famous botanist and the Dean of Fudan University’s graduate school, passed away in a tragic car accident while on a business trip.

Sorrow quickly spread throughout the entire campus of Fudan University, as well as beyond China. Whoever heard the news mourned with a heavy heart. We all have lost a close friend, a wonderful teacher, a mentor, a passionate dreamer and an excellent pioneer in many fields.

**Figure 2:**
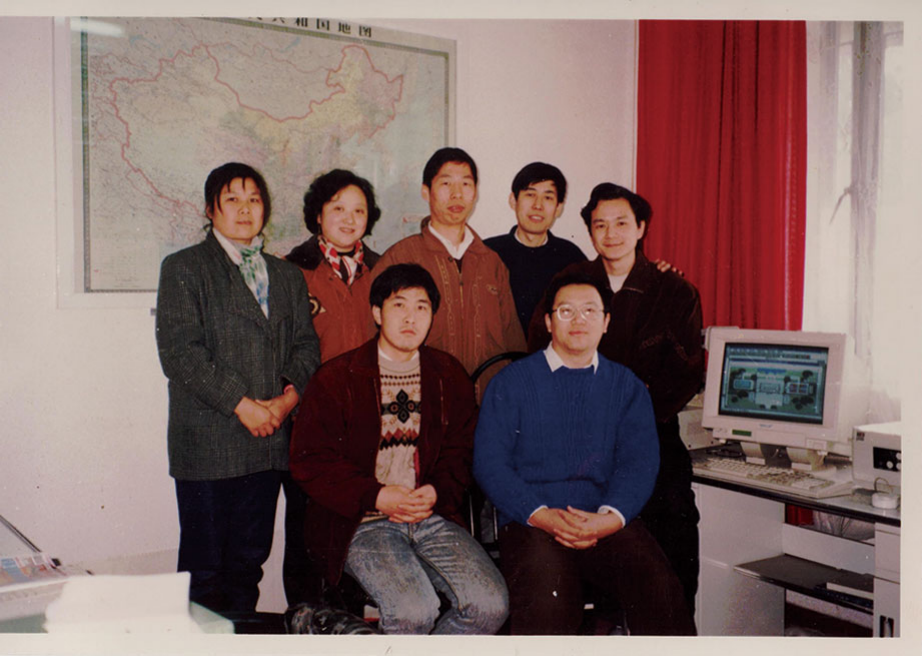
**Youth Laboratory of Computational Biology, Wuhan Insitute of Botany, CAS (1993)**

Born in 1964, in Huanggang, Hubei Province, Yang was marked with the word “early” throughout his life. He entered the University of Science and Technology of China when he was only 15 years old. After graduating from college with a major in radio-electronic engineering, Yang entered the Wuhan Institute of Botany of Chinese Academy of Sciences and started from zero knowledge in the field of botany. With his extraordinary talent and sharp thinking, he started to explore the succession and evolution of plants from a mathematical point of view (钟扬,何芳良, 1986). Inspired by “computational physics” and “computational chemistry”, he put forward the notion of “computational biology”, which he dedicated his energy as a future direction. Yang founded the first computational biology lab at the Wuhan Institute of Botany of Chinese Academy of Sciences in 1993 (Fig. 2).

Together with a group of young people who shared the same view, Yang started a new type of exploration in botany (钟扬,张晓艳, 1990, 钟扬, 1995) and opened up a brilliant chapter of Computational biology in China. Under his leadership, the lab quickly made many great achievements in quantitative taxonomy, branch taxonomy, quantitative ecology, and plant databases which amount to the best in the world. For example, he and Professor Jiakuan Chen were the first ones in China to use quantitative classification and branch classification methods for aquatic plants (钟扬,陈家宽,黄德世, 1990, 钟扬,李伟,黄德世, 1994). However, the concept of “computational biology” was too far ahead of it’s time to be accepted by everyone in China. Instead, people preferred to call it “the application of computers in biology.” Nevertheless, Yang’s work was in line with trends internationally and the first volume of the international academic journal “*Computational Biology*” was published in 1994 and the International Society for Computational Biology (ISCB) was established in 1997. Computational biology has become an inseparable part of basic life sciences in both China and the world. It was entirely fitting that he was later appointed to the Editorial Board of the journal *Computational Biology and Chemistry*.

Yang also greatly advanced the international cooperation in computational biology in the field of botany. At that time, many different computational methods were developed by different scientists in the world, creating a problem of incongruence in taxonomic database systems. In collaboration with the partners from MSU and UC-Berkeley, Yang presented a new interactive classification data model (UNIC structure) to manage hierarchical classification data, and proposed a general comparison methodology for different classification trees and various types of dendrograms (Zhong et al., 1996, 1997). He also designed a prototype taxonomic database system called HICLAS (hierarchical classification system) based on taxonomic onotology (Zhong et al., 1999). Those methods proposed a solution to break the boundaries of different institutions, provided a “lingua franca” for different systems, and laid the foundations for international cooperation. Frank A. Bisby, professor at the University of Reading in the United Kingdom, a leading researcher in taxonomy and bioinformatics, quoted Yang’s operative model in his review paper on biodiversity informatics in *Science* (Bisby, 2000), followed by Yang’s commentary in *Science* in the same year (Zhong et al., 2000).

Another good example is Yang’s collaboration with Prof. Tao Sang of Michigan State University in the field of plant evolution. They proposed a new statistical test model to distinguish hybridization and other biological processes as causes of topological incongruence. The computer simulation analyses they conducted not only supported the validity of the bootstrap test when each gene evolved at a constant rate, but also suggested that the model remained valid as long as the rate heterogeneity was occurring proportionally in the same taxa for both genes (Sang and Zhong, 2000). This work was referred to by Professor M. A. Ragan of the University of Queensland, Australia as one of the advanced aspects in phylogenetics. Collaborating with Professor Suhua Shi and her group in Sun Yat-sen University, Prof. Yang Zhong completed a series of studies on plant phylogenetics. They also detected evolutionary rate heterogeneity among the mangrove genera and their close terrestrial relatives of Rhizophoraceae, using phylogeny analysis and the relative-rate test. Basing on the results of the relative-rate test, the divergence times among genera as well as the average divergence time between the close related inland genus *Carallia* and the coastal Rhizophoreae tribe were determined. These findings were published in *Ecology Letters*, which was the first work completed independently by Chinese scientists published in this internationally recognized peer-reviewed journal in ecology and biodiversity (Zhong et al., 2002).

Yang is well known for his profound knowledge and unique insights, which promoted his collaborations with multidisciplinary experts in China and abroad in the past 10 years. Yang’s expertise and insightful thoughts allowed him to make great contributions to the new frontiers of large-scale genomic and proteomic analysis, molecular evolution analysis of the SARS coronavirus (The Chinese SARS Molecular Epidemiology Consortium, 2004) and genome-wide phylogenetic analysis of *Schistosoma japonicum* to name but two (The Schistosoma japonicum Genome Sequencing and Functional Analysis Consortium, 2009). He also established many early molecular databases in China, including a protein informatics system (the multi-protein survey system, MPSS) (Hao et al., 2005), a database system for plant resistance candidate genes (PlantQTL-GE) (Zeng et al., 2007), and a mouse mutagenesis database, generated with PB transposon methods (PBmice) (Sun et al., 2008). He pioneered computational simulation in plant systems biology to analyze the photosynthetic metabolism of C3 plants, and his work revealed that metabolic pathways are synchronized and coordinated when under environmental perturbations (Luo et al., 2009). This is particularly relevant when organisms adapt to extreme environments, and potentially in understanding how organisms can mitigate against aspect of climate change.

**Figure 3:**
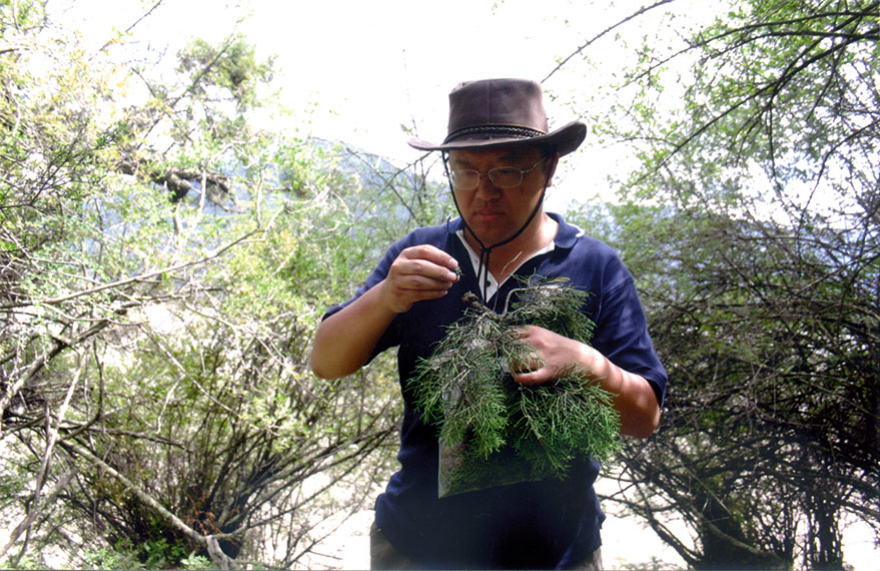
**Prof. Zhong is working on collection and investigation of plant resources**

Yang was also a great educator, and he recognized very early the urgency and importance of cultivating talented Tibetans. Yang was committed to this purpose, and applied for three consecutive periods of nine years as one of the cadres sent to support Tibet. He personally carried on this goal in Tibet for 16 years. Just before his tragic death, he had already booked tickets to go back to work in Tibet University. His health was deteriorating due to the harsh environment of Tibet and frequently traveling between high and low altitudes. There is no doubt that his efforts made tremendous changes in Tibet University. With his help, Tibet University successfully got the first NSFC funding in university history. He trained the first Tibetan PhD in Botany. Yang also made great efforts to establish the very first Masters in Science program (Biology) and the very first PhD program (Ecology) in Tibet University. The ecology department led by Yang also recently entered the pool of the nation’s top disciplines. He conducted a large number of field scientific expeditions and studies in Tibet each year. Significant progress has been made in the study of the genetic diversity and chemical diversity of wild resources in Tibet (Liu et al., 2006; Zhu et al., 2009). Hard work pays off; Yang and his team found the first *Arabidopsis* population in Tibet at an altitude over 4000 m above sea level. As a unique ecotype, Tibet Arabidopsis grows at highest altitude found for the plant, and provides new resources for botany research worldwide. Based on the whole genome sequencing data of this Tibetan Arabidopsis, Yang and his team analyzed the functional genes for high altitude adaptive evolution. Besides Tibet Arabidopsis, Yang and his team also obtained a series of achievements in plant genome variation and adaptive evolution in the extreme environment of the Tibetan Plateau, including the micro-evolution of sea buckthorn and mountain ephedra, the WGS and transcriptome analysis of Qinghai-Tibet cordate houttuynia (Fig. 3) (Qiao et al., 2016). But it wasn’t just plants that inspired him; his group also published a paper on why the Giant Panda eats bamboo (Jin et al., 2011).

Yang had great concerns for the declining quality of domestic education in recent years. While serving as the dean of the Fudan University graduate school, he promoted a number of education-related reforms and launched innovative projects such as the FIST program, which have won praise from all quarters. He believed that education is a “relay race”. Students’ innovative and critical thinking should start from basic education and develop early in childhood. With this sense of responsibility, he devoted a great deal of energy to scientific training and thinking programs for primary and middle school students, adding a heavy burden to his already busy workload. Just before his death he arranged for Prof. James Crabbe, of the Universities of Oxford and Bedfordshire in the UK, and his research collaborator for over 10 years, to visit Qindao and lecture to hundreds of young people—including one of his sons— about science and education. He was keen on public education and science popularization, and translated nearly a dozen books, including the popular seller “*The Great Influenza*: *The Epic Story of the Deadliest Plague in History*”. By translating these excellent books, he spread the seeds of thinking, extending the ideas of education to everyday life.

Professor Zhong Yang’s tragic death is a great loss, not only for the field of plant biology, but also for education as a whole. He has left his beloved research and education career, however his spirits of exploration will never stop; they will carry on in his colleagues, in his students, and in people; his work will inspire in the future. As a scholar, an educator, a pioneer, Professor Yang Zhong will always be in our hearts. His passion will always encourage those who follow his footsteps! As he said, “It is not excellent people who have dreams, but the people who have dreams can be excellent”.

Rest in peace, Dr. Yang Zhong, our best friend, teacher, mentor, and an excellent dreamer! “As long as our heart is flying, the road will continue to extend forwards”.
